# Real‐world efficacy of transfusion with liberal or restrictive strategy in traumatic brain injury

**DOI:** 10.1002/acn3.52272

**Published:** 2024-12-27

**Authors:** Liang‐Wen Cui, Nian Liu, Chao Yu, Ming Fang, Rui Huang, Cheng Zhang, Min Shao

**Affiliations:** ^1^ Department of Critical Care Medicine The First Affiliated Hospital of Anhui Medical University Hefei Anhui China; ^2^ Department of Biostatistics Anhui Provincial Cancer Institute, The First Affiliated Hospital of Anhui Medical University Hefei Anhui China

## Abstract

**Objective:**

The short‐term efficacy of red blood cell (RBC) transfusion among general traumatic brain injury (TBI) patients is unclear.

**Methods:**

We used the MIMIC database to compare the efficacy of liberal (10 g/dL) versus conservative (7 g/dL) transfusion strategy in TBI patients. The outcomes were neurological progression (decrease of Glasgow coma scale (GCS) of at least 2 points) and death within 28 days of ICU admission. Each eligible individual was cloned and assigned each of the replicates to one of the treatment arm. The imbalance induced by informative censoring was adjusted by inverse probability weighting. The standardized, weighted pooled logistic regression with 500 bootstrap resampling was used to estimate the cumulative risk difference and 95% confidence interval (CI).

**Results:**

Of the 1141 eligible individuals, 29.0% received RBC transfusion. Compared with the restrictive group, the liberal strategy reduced early death (3 days: 5%, 95% CI: 2%–7%; 7 days: 6%, 95% CI: 3%–11%); however, no significant difference of mortality risk at 28‐day or neurological progression risk at any time points was observed. The risk of coagulopathy at 3 days was increased by 7% (95% CI: 1%–19%) in the liberal group. The subgroup analysis indicated a beneficial effect of liberal transfusion on mortality in hemodynamically unstable patients.

**Interpretation:**

Compared with the restrictive strategy, the liberal strategy does not improve the short‐term neurological prognosis and death among patients with TBI in a real‐world situation. The liberal strategy may be beneficial to survival at very early stage or in hemodynamically unstable subgroup.

## Background

Traumatic brain injury (TBI) is a leading cause of neurological dysfunction worldwide.^1^ Anemia is common among TBI patients,^2^ and is probably associated with poor outcomes due to tissue hypoxia and metabolic crises induced by low hemoglobin (Hb) levels.^3^, ^4^ Red blood cell (RBC) transfusion is frequent in patients with TBI and anemia^5^; however, target Hb level is not clearly determined in this population. The wide variation in transfusion practices across different studies highlights the lack of clinical evidence to guide transfusion strategies for TBI,^5^ which results in the diversity of physicians' responses to the Hb thresholds for RBC transfusion after TBI in clinical practice.^6^, ^7^

The optimal transfusion threshold has been investigated retrospectively,^8^, ^9^ and the results should be interpreted with caution due to study heterogeneity and inherent flaw in observational studies, which urges the need for evidence from randomized controlled trial (RCT). To date, three RCTs^4^, ^10^, ^11^ have included patients with moderate or severe TBI and demonstrated inconsistent conclusions regarding the efficacy of “restrictive” versus “liberal” transfusion strategy on 6‐month neurological outcome and mortality. The most recent pragmatic trial (also the largest trial by far, *N* = 742), the HEMOTION trial,^11^ has reported no significant difference of 6‐month outcomes between the liberal (10 g/dL) and conservative (7 g/dL) transfusion strategies. The RESTRIC trial includes severe TBI patients and compares the restrictive and liberal transfusion strategies (target hemoglobin levels of 7–9 g/dL and 10–12 g/dL, respectively). The results show that the non‐inferiority of restrictive transfusion to liberal transfusion for 28‐day survival is not statistically significant.^12^ No published or registered ongoing RCTs aim to compare the efficacy of different transfusion strategies on in‐hospital outcomes (e.g., clinical progression or death) among the whole TBI population with diverse severity. To address this issue and to make a causal inference of the treatment effect from real‐world data, we emulate a hypothetical pragmatic randomized trials (e.g., the target trial) that would answer the question aforementioned. This framework avoids erroneous casual conclusions by preventing biases that are commonly encountered in observational analyses.^13^ We use the Medical Information Mart for Intensive Care (MIMIC) database to emulate a pragmatic trial that compares the short‐term efficacy of different RBC transfusion strategies in TBI.

## Methods

### Data source

MIMIC‐IV is a contemporary electronic health record dataset covering a decade of admissions of Beth Israel Deaconess Medical Center and Massachusetts Institute of Technology between 2008 and 2019.^14^ We used version 2.2 which includes 431,231 admissions and 73,181 ICU stays. The dataset provides baseline characteristics, mortality and granular information of measurements and treatments, which makes possible to emulate a target trial in this study.

### Study design and eligible criteria

We conducted a pragmatic clinical trial by applying the target trial emulation framework with cloning‐censoring‐weighting approach. The specification and emulation of the target trial are presented in Table S1. The inclusion criteria were as follows: patients who were admitted to hospital due to TBI, aged 18 years or older, and Hb < 10 g/dL within 7 days from ICU admission. The diagnosis of TBI at hospital admission was extracted by ICD codes.^15^ The exclusion criteria were pregnant, hemorrhagic shock, GCS of 3, and non‐reactive pupils. We did not exclude patients who had received RBC transfusion before ICU admission. T0 was defined as the first time of being eligible after ICU admission. All patients were followed up from T0 until an occurrence of the outcomes, transfer out, or 28 days after T0, whichever occurred first.

### Treatment strategies

We compared the effect of RBC transfusion strategies (liberal vs. restrictive) in TBI patients. Liberal transfusion strategy was defined as receiving RBC transfusion when Hb < 10 g/dL during the grace period. Restrictive transfusion strategy was defined as receiving RBC transfusion when Hb <7 g/dL during the grace period. The transfusion thresholds were selected on the basis of available evidence.^11^ The grace period was defined as 24 h after the prespecified threshold was reached in the main analysis.

### Study outcomes

The study outcomes were clinical progression, which was defined as a decrease of GCS by at least 2 points, and all‐cause mortality. The safety outcomes were any suspected infection, acute respiratory distress syndrome (ARDS, defined as PaO2/FiO2 < 300), sepsis/septic shock (defined as organ failure and suspected infection, usually implemented as SOFA≥2 and proximal antibiotics/cultures), and coagulopathy (defined as a prothrombin time international normalized ratio greater than 1.4 or platelet count less than 100,000 mm^3^).^10^

### Covariates

Source of bias was illustrated by a directed acyclic graph^16^ (Fig. S2) based on previous literature and clinical knowledge.^8^, ^17^, ^18^, ^19^, ^20^, ^21^, ^22^ The confounders, which were causes of both the treatment strategy and the outcome, included age, sex, Hb, GCS, mean arterial pressure (MAP), lactate (<2, 2–4, 4+, unknown), blood urea nitrogen (BUN), intracranial pressure (ICP) (≤20, 21–40, 40+, unknown), prior vasopressors prescription, hemorrhage, ischemic heart disease, and multiple injuries. Missing data on those variables were imputed by the last observation carried forward method based on the assumption that there variables remained stable during the interval between two measurements. All diagnoses were identified by ICD codes (Table S2).

### Statistical analysis

We applied cloning, censoring, and weighting approach to emulate a target trial.^23^, ^24^ We created a dataset with two clones of each eligible individual and assigned each of the replicates to one of the treatment arm at T0. Thereafter, at hourly intervals, we assessed whether replicates adhered to their assigned arm by artificial censoring; replicates were censored if and when their actual treatment deviated from their assigned treatment strategy. Specifically, if a replicate was assigned to liberal strategy, but did not receive RBC transfusion by the end of 24 h, they would be censored at that point; if the replicate received RBC transfusion or reached the endpoint within the grace period, they remained uncensored. Conversely, if a replicate was assigned to restrictive strategy, but received transfusion when Hb ≥7.0 g/dL or did not receive RBC transfusion within 24 h after Hb <7.0 g/dL, they would be censored at transfusion or grace period; if the replicate received treatment or reached the endpoint within 24 h after Hb <7.0 g/dL, or Hb ≥7.0 g/dL with no transfusion treatment, they remained uncensored.

To balance the selection bias induced by informative censoring, each individual received a time‐varying inverse probability weight (IPW), which was the reverse of the probability that a replicate remained uncensored conditional on baseline characteristics.^25^, ^26^ The weights were estimated by fitting a pooled logistic model for the hourly probability of remaining uncensored, including variables for time (linear and quadratic forms) and the baseline covariates aforementioned, and truncated at the 99th percentile to avoid influence of extreme values.

We estimated the effect of liberal transfusion strategy using standardized, weighted pooled logistic regression (a discrete‐time hazard model), including treatment strategy, time (linear and quadratic forms), and their interactions to allow for non‐proportional hazards.^27^ The predicted probabilities from this model were used to estimate the adjusted predicted probability of the outcomes under each treatment strategy and produce standardized, weighted survival probability curves. We estimated the 3‐, 7‐, 14‐, and 28‐day absolute risks and risk differences with 95% confidence intervals (CIs) using a non‐parametric bootstrap of 500 samples on patient level. Risks were considered to be statistically significant when the 95% CIs did not cross zero.

Baseline characteristics before and after weighting were presented as median (IQR) for continuous variables and as numbers (%) for categorical variables. Standardize mean difference (SMD) was used to evaluate the balance of patient characteristics between two treatment groups. An SMD of <0.1 is considered of good balance.

Several sensitivity analyses were performed to test the robustness and consistency of our results. Firstly, we modified restrictive strategy threshold from 7.0 g/dL to 7.5, 8.0 g/dL to test the effect of different thresholds. Secondly, we extended the grace period from 24 to 48 and 72 h to test the impact of being timely transfused. Thirdly, we compared the results using the doubly robust model by adjusting for the IPWs and all baseline covariates.^28^ Fourthly, we performed some subgroup analyses to explore the potential heterogeneity of the treatment. All statistical analyses were carried out using R (version 3.4.0).

## Results

A total of 1141 eligible individuals were included (Fig. S1) and Table 1 shows the baseline characteristics of the study population. Ninety‐two per cent of the total patients were with mild TBI. The Hb level apparently declined within 7 days after ICU admission; 18.2% of the patients had a lowest Hb level of below 7.0 g/dL within this period. Three‐hundred and thirty individuals (29.0%) received RBC transfusion and the median time to initiate transfusion was 18 [interquartile range (IQR): 4.0, 41.8] h after ICU admission.

**Table 1:** Baseline characteristics of the study population.

Variable	Description
Age, median (IQR)	68.0 (50.0, 82.0)
≥65 years, *N* (%)	624 (54.7)
<65 years	517 (45.3)
Sex, *N* (%)	
Female	635 (55.7)
Male	506 (44.3)
GCS, median (IQR)	15.0 (14.0, 15.0)
>12, *N* (%)	1050 (92.0)
≤12	91 (8.0)
GCS motor, *N* (%)	
6	688 (60.2)
5	235 (20.6)
4	84 (7.4)
3	13 (1.1)
2	15 (1.3)
1	106 (9.4)
Baseline Hb, median (IQR)	9.3 (8.8, 9.7)
≥7.0 g/dL, *N* (%)	1102 (96.6)
<7.0 g/dL	39 (3.4)
Lowest Hb within 7 days, median (IQR)	8.2 (7.2, 9.1)
≥7.0 g/dL, *N* (%)	933 (81.7)
<7.0 g/dL	208 (18.2)
RBC transfusion, *N* (%)	
Yes	330 (29.0)
No	811 (71.0)
RBC transfusion time, hours, median (IQR)	18.0 (4.0, 41.8)
BUN, mg/dL, median (IQR)	16.0 (11.0, 25.0)
MAP, mmHg, median (IQR)	79.0 (70.0, 89.0)
Lactate, *N* (%)	
<2 mmol/L	465 (40.8)
2–3.9 mmol/L	208 (18.2)
4+ mmol/L	73 (6.4)
Unknown	395 (34.6)
ICP, *N* (%)	
<15 mmHg	49 (4.3)
15–20 mmHg	13 (1.1)
21–40 mmHg	15 (1.3)
41+ mmHg	2 (0.2)
Unknown	1062 (93.1)
Vasopressors, *N* (%)	
Yes	312 (27.3)
No	829 (72.7)
Active bleeding, *N* (%)	
Yes	7 (0.6)
No	1134 (99.4)
Ischemic heart disease, *N* (%)	
Yes	210 (18.4)
No	931 (81.6)
Multiple injuries, *N* (%)	
Yes	626 (54.9)
No	515 (45.1)

BUN, blood urea nitrogen; GCS, Glasgow coma scale; Hb, hemoglobin; ICP, intracranial pressure; IQR, interquartile range; MAP, mean arterial pressure; RBC, red blood cell.

Of the 2282 replicates in the expanded dataset, 194 (17.0%) of the restrictive group and 806 (70.7%) of the liberal group were censored during follow‐up in the progression analysis. In the mortality analysis, the respective numbers were 255 (22.3%) and 935 (82.0%). Among the uncensored, the median (IQR) follow‐up time was 75 (27, 157) h for the restrictive group (120,187 person‐hours) and 19.5 (10.0, 103.3) h for the liberal group (45,907 person‐hours) in the progression analysis. In the mortality analysis, median follow‐up times were 672.0 h for both groups. The estimated IPWs had mean 2.0 (IQR 1.0–1.4, 99th percentile 45.9) for the progresion analysis, and mean 3.1 (IQR 1.1–1.5, 99th percentile 90.3) for the mortality analysis. The main predictors of censoring were Hb, MAP, lactate, vasopressor, multiple injuries and time since T0. The covariants were in good balance after weighting (Fig. S3).

**Figure 1:**
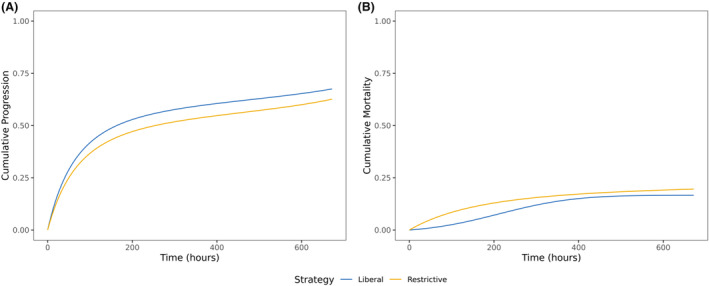
Standardized weighted cumulative hazard curves comparing the efficacy of different transfusion strategies in TBI. (A) Clinical progression assessed by Glasgow comma scale decrease by at least 2 points; (B) all‐cause mortality. TBI, traumatic brain injury.

**Figure 2:**
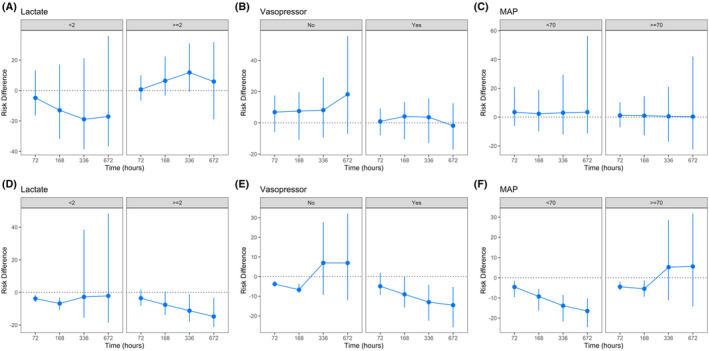
Subgroup analyses of the main outcomes stratified by hemodynamic indicators. (A–C) Clinical progression; (D–F) all‐cause mortality.

There were 385 clinical progression events in the restrictive group, and 256 events in the liberal group (Table 2). Among those who developed progression, the median (IQR) time to the event was 31.0 (12.8, 63.0) h for the restrictive group and 16.0 (1.0, 9.0) h for the liberal group. Among those who died, the median (IQR) time to death was 132.5 (45.8, 240.8) h for the restrictive group and 22.0 (7.0, 145.5) h for the liberal group. Compared with the restrictive group, the 28‐day adjusted standardized ratio differences (95% CI) for were 3.26% (−20.31%, 30.10%) for progression and − 2.97% (−14.03%, 19.61%) for death (Table 2, Fig. 1). Of note, liberal strategy significantly reduced early death by 5% at 3 days and 6% at 7 days. Adjustment for either grace time or transfusion threshold did not materially change the significance of the main results (Table S3); however, modification of the grace period resulted in a more apparent change of the cumulative hazard curves (Fig. S4). The doubly robust approach revealed a similar result (Table S4). The subgroup analysis revealed that liberal transfusion might be beneficial for mortality during the whole course in hemodynamically unstable patients (Fig. 2 and Table S5).

**Table 2:** Main outcomes of the ETT that compares the efficacy of different transfusion strategies in TBI.

	Restrictive	Liberal
Clinical progression		
Number of observation (person‐hours)	120,187	45,907
Number of event	385	256
Number of censoring	194	806
Cumulative risk difference (%)		
3‐day	–	2.91 (−6.53, 10.15)
7‐day	–	4.59 (−10.83, 14.31)
14‐day	–	5.40 (−15.20, 20.15)
28‐day	–	3.26 (−20.31, 30.10)
All‐cause mortality		
Number of observation (person‐hours)	530,351	122,798
Number of event	171	67
Number of censoring	255	935
Cumulative risk difference (%)		
3‐day	–	−5.02 (−7.51, −2.40)
7‐day	–	−6.27 (−11.22, −3.00)
14‐day	–	−2.34 (−11.80, 14.56)
28‐day	–	−2.97 (−14.03, 19.61)

ETT, emulated target trial; TBI, traumatic brain injury.

**Figure 3:**
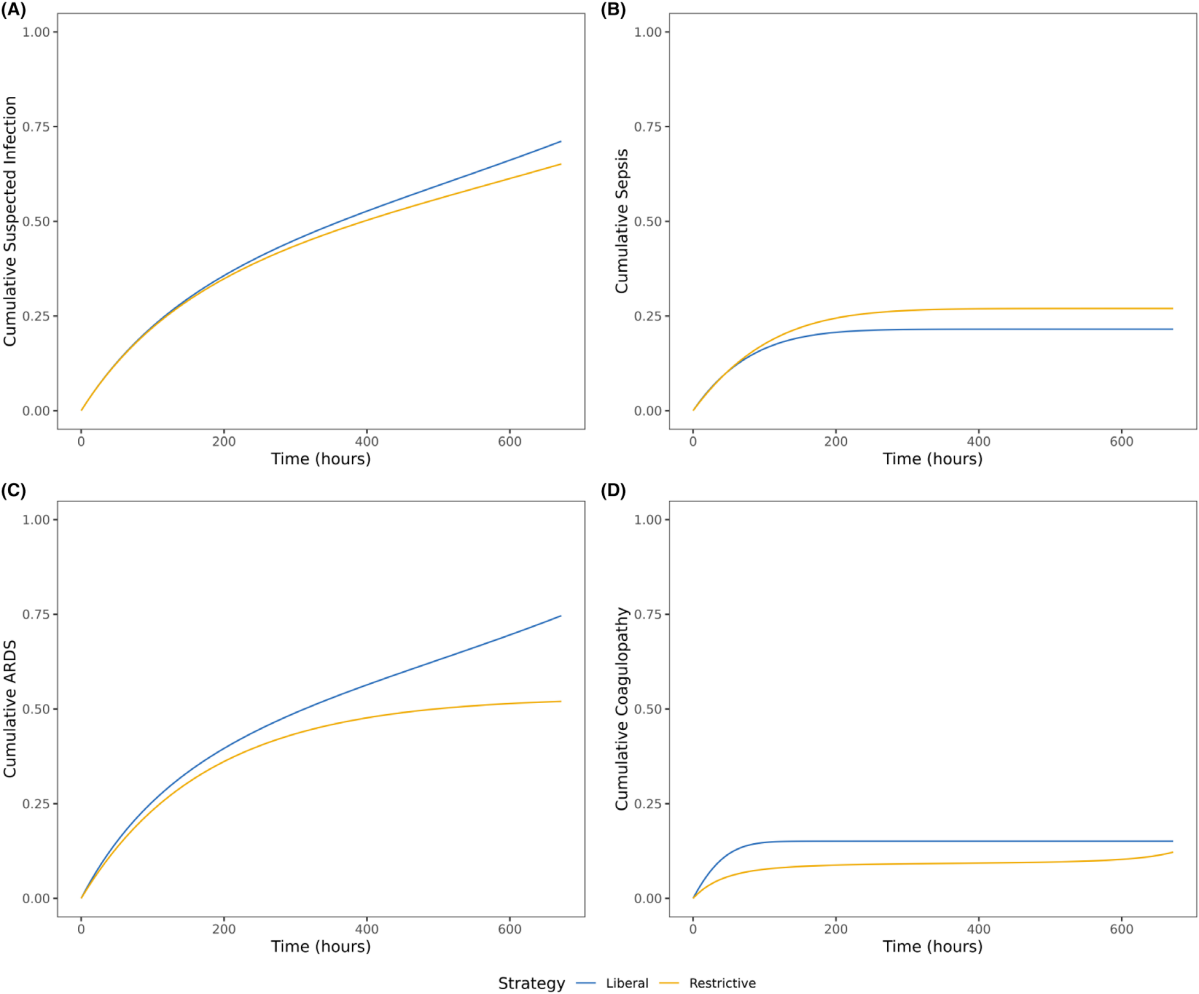
Standardized weighted cumulative hazard curves comparing the safety of different transfusion strategies in TBI. (A) Any suspected infection; (B) sepsis; (C) ARDS; (D) coagulopathy. ARDS, acute respiratory distress syndrome; TBI, traumatic brain injury.

For safety analysis, we observed that the risk of early coagulopathy was increased in the liberal group, showing a 3‐day risk difference (95% CI) as 6.86% (0.72%, 18.62%) (Table 3). No risk difference at the pre‐specified time points was observed for infection, ARDS, and sepsis (Table 3, Fig. 3, Table S5).

**Table 3:** Risk difference (%) of the safety outcomes in the ETT comparing liberal versus restrictive strategy for TBI.

Time	Infection	Sepsis	ARDS	Coagulopathy
3‐day	0.19 (−5.05, 12.32)	−0.52 (−4.56, 8.44)	1.93 (−5.42, 9.96)	6.86 (0.72, 18.62)
7‐day	0.65 (−11.14, 19.79)	−3.06 (−9.37, 11.13)	3.01 (−10.36, 19.15)	6.51 (−0.15, 17.99)
14‐day	2.21 (−10.64, 34.13)	−5.33 (−13.13, 10.89)	7.92 (−16.81, 28.98)	5.83 (−0.74, 16.79)
28‐day	6.00 (−11.74, 39.88)	−5.43 (−13.43, 12.30)	22.61 (−2.41, 45.59)	2.90 (−4.02, 14.94)

ARDS, acute respiratory distress syndrome; ETT, emulated target trial; TBI, traumatic brain injury.

## Discussion

In our study, more than 90% of patients were mild TBI, and anemia occurred in about one‐third of the total population. There were no statistically significant differences between restrictive and liberal transfusion strategies in neurological recovery and mortality at 28‐day, whereas the liberal strategy was associated with a decrease in mortality at 3 and 7 days, and the results of subgroup and sensitivity analyses were consistent with the main analysis. The liberal strategy was associated with an increased incidence of coagulopathy as an adverse event at 3‐day after transfusion.

The results of our study showed that the liberal transfusion group was protective against very early all‐cause mortality compared with the restricted group, but the protective effect disappeared afterwards. Interestingly, the liberal strategy was protective in the hemodynamically unstable subgroup. Current studies suggest that TBI‐related hemodynamic instability is commonly induced by the initial primary brain injury, during which mechanical forces lead to axonal shearing and necrosis, followed by secondary injury driven by inflammation, blood–brain barrier disruption, apoptosis, metabolic disturbances, and oxidative stress.^29^, ^30^, ^31^, ^32^ The cascade of autonomic and inflammatory mediators released into the circulation leads to changes in central and peripheral autonomic tone, the hypothalamic–pituitary–adrenal axis and the sympathetic nervous system leading to compromised release of a wide range of catecholamines,^33^ peripheral vasodilatation, generation of capillary leakage,^34^, ^35^ lack of effective volume, and varying degrees of circulatory involvement. Patients with TBI are associated with varying degrees of blood loss. The use of dehydrating agents results in insufficient effective circulating blood volume. All of these mechanisms may lead to circulatory instability in patients with TBI. As the body continues to compensate, vascular tone improves, capillary leakage improves, and blood volume is corrected; so that the liberal transfusion group has a protective effect on all‐cause mortality at very early phase, but this protection disappears after 14 and 28 days. During resuscitation from TBI, crystalloid fluid extravasates across the compromised blood–brain barrier, which significantly increases the water content of the brain and leads to cytotoxic edema that can be alleviated by increasing plasma colloid osmolality.^36^ Thus the liberal transfusion strategy may show a protective effect against death when TBI patients are hemodynamically unstable.

TBI has short‐ and long‐term adverse clinical outcomes, including death and disability. Anemia was common in patients with TBI and was associated with an increased risk of poor outcomes,^37^ so blood transfusion is considered an important intervention in this population.^38^ A study randomly selected 200 patients with closed head injury and found no significant difference in neurological recovery at 6 weeks with restrictive (Hb <7 g/dL) versus liberal (Hb < 10 g/dL) transfusion thresholds.^4^ A secondary analysis found that, compared to the liberal group, the restrictive group had more episodes of brain tissue hypoxia (cerebral tissue oxygenation <10 mm Hg) in normal brain tissue, but there were no differences in intracranial pressure, long‐term neurological outcomes, or mortality.^39^ Similarly, in the TRICC trial subgroup analysis, there was no difference in 30‐day mortality for patients with moderate to severe TBI.^40^

Updated clinical guidelines and reviews comparing the effects of liberal versus restrictive transfusion strategies have emphasized that the available data are insufficient to guide transfusion practice in patients with TBI.^41^, ^42^ A review of RBC transfusion strategies in the ICU,^43^ with substantial clinical research evidence supporting the use of restrictive transfusion strategy compared with more liberal strategy. In recent years, the number of RCTs comparing restrictive versus liberal transfusion thresholds has increased significantly, and it is generally considered that a Hb level of 7–8 g/dl is considered restrictive, and a Hb level of 9–10 g/d is considered a liberal transfusion threshold.^44^ In summary, the restrictive transfusion threshold is not inferior to the liberal transfusion threshold on mortality and adverse effects.^44^ However, for certain subgroups, such as for patients with TBI, especially mild TBI, the data are insufficient to develop reliable recommendations. A number of researches have been conducted on the prognostic aspects of blood transfusion strategies in moderate to severe TBI,^2^, ^11^, ^22^, ^37^, ^45^, ^46^ whereas in mild TBI, the main focus of current research is on the aspects of post‐injury mental health assessment and psychological disorders,^47^, ^48^ and there are almost no studies on the threshold of RBC transfusion.

Two recent trials on transfusion thresholds, HEMOTION (NCT03260478)^11^ was a large RCT of patients with moderate or severe TBI, designed to compare the restrictive transfusion threshold (Hb 7–8 g/dl) versus liberal transfusion threshold (Hb 9–10 g/dl) on prognosis. The results suggested that the restrictive transfusion strategy is not inferior to the liberal transfusion strategy in some aspects, yet the mild TBI population was outside the scope of this study. Another large, multicenter, prospective, observational study of TBI patients in Europe, CENTER‐TBI (NCT02210221) was a secondary analysis to assess the relationship between Hb values and long‐term prognosis in critically ill TBI patients.^46^ In the second paragraph of the discussion, a large section describes the mechanism of transfusion benefit in TBI patients, and it is very regrettable that the current study did not go into the breakdown of the mechanism of transfusion benefit in patients with mild, moderate, and severe TBI. Therefore we look forward to more in‐depth studies at the cellular molecular level. The HEMOTION trial included patients with moderate or severe TBI; however, the number of patients with mild TBI is significantly higher among all TBI patients, and research for this group of patients is much more meaningful, and our study fills a gap in this area. There was a considerable heterogeneity across centers with respect to threshold transfusion, focusing on the relationship between anemia and long‐term adverse neurological prognosis and mortality in critically ill TBI patients, and only bundled descriptions of the impact of transfusion thresholds. In this study, we included a very general population including all TBI patients without specific refinement of the transfusion threshold situation for different degrees of injury, which aimed to bridge the gap in transfusion strategies for patients with mild TBI. One large‐scale ongoing randomized trial^49^ exams two different Hb thresholds to initiate RBC transfusions in this setting and will provide important information for managing anemic TBI patients. In a systematic review of studies in neurocritical care patients, there are insufficient evidence to support the use of a specific transfusion threshold to improve morbidity and mortality.^20^ Unfortunately, the literature available on transfusion strategies for patients with mild TBI is not as comprehensive as needed, our study could augment this knowledge.

This study had several limitations. Firstly, randomization was simulated by statistical methods, therefore unmeasured confounders would have a substantial effect. However, this emulated target trial is conducted on a large‐scale real‐world dataset, making the findings more relevant to real‐world situations. We hoped that subsequent high‐quality RCTs will be conducted. Secondly, our data originate from a developed country, thus the generalizability of our results to different populations may be limited.

## Conclusions

This emulated target trial suggests that, compared with the restrictive RBC transfusion strategy, the liberal strategy does not improve the short‐term neurological prognosis and death among patients with TBI in a real‐world situation. The liberal strategy may be beneficial to survival at very early stage or in hemodynamically unstable subgroup, which needs to be confirmed by further clinical trials.

## Funding Information

National Natural Science Foundation of China (82370605), China Postdoctoral Science Foundation, and the 74th batch of top‐level funding projects (2023M740029).

## Conflict of Interest

The authors declare that they have no competing interests.

## Author Contributions

Liang‐Wen Cui and Cheng Zhang analyzed the data. Liang‐Wen Cui, Nian Liu, Chao Yu, Ming Fang, Rui Huang, and Min Shao interpreted the data. Liang‐Wen Cui and Cheng Zhang drafted the work. All authors conceived, designed, and revised the work. All authors read and approved the final manuscript.

## Consent for Publication

Not applicable.

## Supporting information


Data S1.

## Data Availability

The datasets generated and/or analyzed during the current study are available in https://physionet.org/content/mimiciv/2.2/.

## References

[1] Dewan MC , Rattani A , Gupta S , et al. Estimating the global incidence of traumatic brain injury. J Neurosurg. 2019;130(4):1080‐1097. 29701556 10.3171/2017.10.JNS17352

[2] Salim A , Hadjizacharia P , DuBose J , et al. Role of anemia in traumatic brain injury. J Am Coll Surg. 2008;207(3):398‐406. 18722946 10.1016/j.jamcollsurg.2008.03.013

[3] Yamal J‐M , Benoit JS , Doshi P , et al. Association of transfusion red blood cell storage age and blood oxygenation, long‐term neurologic outcome, and mortality in traumatic brain injury. J Trauma Acute Care Surg. 2015;79(5):843‐849. 26496111 10.1097/TA.0000000000000834 PMC4621763

[4] Robertson CS , Hannay HJ , Yamal J‐M , et al. Effect of erythropoietin and transfusion threshold on neurological recovery after traumatic brain injury. JAMA. 2014;312(1):36‐47. 25058216 10.1001/jama.2014.6490 PMC4113910

[5] Boutin A , Chassé M , Shemilt M , et al. Red blood cell transfusion in patients with traumatic brain injury: a systematic review and meta‐analysis. Transfus Med Rev. 2016;30(1):15‐24. 26409622 10.1016/j.tmrv.2015.08.004

[6] Lessard Bonaventure P , Lauzier F , Zarychanski R , et al. Red blood cell transfusion in critically ill patients with traumatic brain injury: an international survey of physicians' attitudes. Can J Anesth. 2019;66(9):1038‐1048. 31012052 10.1007/s12630-019-01369-w

[7] Sena MJ , Rivers R , Muizelaar J , Battistella F , Utter G . Transfusion practices for acute traumatic brain injury: a survey of physicians at US trauma centers. Intensive Care Med. 2009;35(3):480‐488. 18854976 10.1007/s00134-008-1289-z

[8] Komurcu O , Dost B , Ozdemir E , Aras M , Ulger F . Red blood cell transfusion and hemoglobin level on neurological outcome in the first 24 hours of traumatic brain injury. Am J Emerg Med. 2022;59:74‐78. 35809538 10.1016/j.ajem.2022.06.058

[9] Moman RN , Kor DJ , Chandran A , et al. Red blood cell transfusion in acute brain injury subtypes: an observational cohort study. J Crit Care. 2019;50:44‐49. 30471560 10.1016/j.jcrc.2018.11.006 PMC6381596

[10] Gobatto ALN , Link MA , Solla DJ , et al. Transfusion requirements after head trauma: a randomized feasibility controlled trial. Crit Care. 2019;23(1):89. 30871608 10.1186/s13054-018-2273-9 PMC6419414

[11] Turgeon AF , Fergusson DA , Clayton L , et al. Liberal or restrictive transfusion strategy in patients with traumatic brain injury. N Engl J Med. 2024;391:722‐735. 38869931 10.1056/NEJMoa2404360

[12] Hayakawa M , Tagami T , Kudo D , et al. The restrictive red blood cell transfusion strategy for critically Injured patients (RESTRIC) trial: a cluster‐randomized, crossover, non‐inferiority multicenter trial of restrictive transfusion in trauma. J Intensive Care. 2023;11(1):34. 37488591 10.1186/s40560-023-00682-3 PMC10364403

[13] Hernán MA , Sauer BC , Hernández‐Díaz S , Platt R , Shrier I . Specifying a target trial prevents immortal time bias and other self‐inflicted injuries in observational analyses. J Clin Epidemiol. 2016;79:70‐75. 27237061 10.1016/j.jclinepi.2016.04.014 PMC5124536

[14] Johnson AEW , Bulgarelli L , Shen L , et al. MIMIC‐IV, a freely accessible electronic health record dataset. Scientific Data. 2023;10(1):219. 36596836 10.1038/s41597-022-01899-x PMC9810617

[15] Korley FK , Kelen GD , Jones CM , Diaz‐Arrastia R . Emergency department evaluation of traumatic brain injury in the United States, 2009–2010. J Head Trauma Rehabil. 2016;31(6):379‐387. 26360006 10.1097/HTR.0000000000000187 PMC4786477

[16] Tennant PWG , Murray EJ , Arnold KF , et al. Use of directed acyclic graphs (DAGs) to identify confounders in applied health research: review and recommendations. Int J Epidemiol. 2021;50(2):620‐632. 33330936 10.1093/ije/dyaa213 PMC8128477

[17] Ngwenya LB , Suen CG , Tarapore PE , Manley GT , Huang MC . Safety and cost efficiency of a restrictive transfusion protocol in patients with traumatic brain injury. J Neurosurg. 2018;128(5):1530‐1537. 28644101 10.3171/2017.1.JNS162234

[18] Sekhon MS , McLean N , Henderson WR , Chittock DR , Griesdale DE . Association of hemoglobin concentration and mortality in critically ill patients with severe traumatic brain injury. Crit Care. 2012;16(4):R128. 22817913 10.1186/cc11431 PMC3580711

[19] Roberts DJ , Zygun D . Anemia, red blood cell transfusion, and outcomes after severe traumatic brain injury. Crit Care. 2012;16(5): 154. 22979948 10.1186/cc11489 PMC3682251

[20] Desjardins P , Turgeon AF , Tremblay M‐H , et al. Hemoglobin levels and transfusions in neurocritically ill patients: a systematic review of comparative studies. Crit Care. 2012;16(2):R54. 22471943 10.1186/cc11293 PMC3681381

[21] Omar M , Moore L , Lauzier F , et al. Complications following hospital admission for traumatic brain injury: a multicenter cohort study. J Crit Care. 2017;41:1‐8. 28477507 10.1016/j.jcrc.2017.04.031

[22] Vedantam A , Yamal J‐M , Rubin ML , Robertson CS , Gopinath SP . Progressive hemorrhagic injury after severe traumatic brain injury: effect of hemoglobin transfusion thresholds. J Neurosurg. 2016;125(5):1229‐1234. 26943843 10.3171/2015.11.JNS151515 PMC5065393

[23] Hernán MA . How to estimate the effect of treatment duration on survival outcomes using observational data. BMJ. 2018;360:k182. 29419381 10.1136/bmj.k182 PMC6889975

[24] Hernán MA , Robins JM . Using big data to emulate a target trial when a randomized trial is not available: table 1. Am J Epidemiol. 2016;183(8):758‐764. 26994063 10.1093/aje/kwv254 PMC4832051

[25] Hernán MA , Alonso A , Logan R , et al. Observational studies analyzed like randomized experiments. Epidemiology. 2008;19(6):766‐779. 18854702 10.1097/EDE.0b013e3181875e61 PMC3731075

[26] Mansournia MA , Altman DG . Inverse probability weighting. BMJ. 2016;352:i189. 26773001 10.1136/bmj.i189

[27] Hernán MA . The hazards of Hazard ratios. Epidemiology. 2010;21(1):13‐15. 20010207 10.1097/EDE.0b013e3181c1ea43 PMC3653612

[28] Funk MJ , Westreich D , Wiesen C , Stürmer T , Brookhart MA , Davidian M . Doubly robust estimation of causal effects. Am J Epidemiol. 2011;173(7):761‐767. 21385832 10.1093/aje/kwq439 PMC3070495

[29] Gundappa P . Extracranial complications of traumatic brain injury: pathophysiology—a review. J Neuroanaesth Crit Care. 2019;6(3):200‐212.

[30] Toro C , Jain S , Sun S , et al. Association of brain injury biomarkers and circulatory shock following moderate‐severe traumatic brain injury: a TRACK‐TBI study. J Neurosurg Anesthesiol. 2023;35(3):284‐291. 34967764 10.1097/ANA.0000000000000828 PMC9243189

[31] Kinoshita K . Traumatic brain injury: pathophysiology for neurocritical care. J Intensive Care. 2016;4(1):29. 27123305 10.1186/s40560-016-0138-3 PMC4847183

[32] Thapa K , Khan H , Singh TG , Kaur A . Traumatic brain injury: mechanistic insight on pathophysiology and potential therapeutic targets. J Mol Neurosci. 2021;71(9):1725‐1742. 33956297 10.1007/s12031-021-01841-7

[33] Brand J , McDonald SJ , Gawryluk JR , Christie BR , Shultz SR . Stress and traumatic brain injury: an inherent bi‐directional relationship with temporal and synergistic complexities. Neurosci Biobehav Rev. 2023;151:105242. 37225064 10.1016/j.neubiorev.2023.105242

[34] Saravi B , Goebel U , Hassenzahl LO , et al. Capillary leak and endothelial permeability in critically ill patients: a current overview. Intensive Care Med Exp. 2023;11(1):96. 38117435 10.1186/s40635-023-00582-8 PMC10733291

[35] Siddall E , Khatri M , Radhakrishnan J . Capillary leak syndrome: etiologies, pathophysiology, and management. Kidney Int. 2017;92(1):37‐46. 28318633 10.1016/j.kint.2016.11.029

[36] Van Aken HK , Kampmeier TG , Ertmer C , Westphal M . Fluid resuscitation in patients with traumatic brain injury: what is a SAFE approach? Curr Opin Anaesthesiol. 2012;25(5):563‐565. 22825048 10.1097/ACO.0b013e3283572274

[37] Lelubre C , Bouzat P , Crippa IA , Taccone FS . Anemia management after acute brain injury. Crit Care. 2016;20(1):152. 27311626 10.1186/s13054-016-1321-6 PMC4911680

[38] Kramer AH , Zygun D . Anemia and red blood cell transfusion in neurocritical care. Crit Care. 2009;13(3):R89. 19519893 10.1186/cc7916 PMC2717460

[39] Yamal J‐M , Rubin ML , Benoit JS , et al. Effect of hemoglobin transfusion threshold on cerebral hemodynamics and oxygenation. J Neurotrauma. 2015;32(16):1239‐1245. 25566694 10.1089/neu.2014.3752 PMC4532899

[40] McIntyre LA , Fergusson DA , Hutchison JS , et al. Effect of a liberal versus restrictive transfusion strategy on mortality in patients with moderate to severe head injury. Neurocrit Care. 2006;5(1):4‐9. 16960287 10.1385/ncc:5:1:4

[41] Carson JL , Stanworth SJ , Roubinian N , et al. Transfusion thresholds and other strategies for guiding allogeneic red blood cell transfusion. Cochrane Database Syst Rev. 2016;2016(10):CD002042. 10.1002/14651858.CD002042.pub4 PMC6457993 27731885

[42] Carson JL , Stanworth SJ , Guyatt G , et al. Red blood cell transfusion: 2023 AABB international guidelines. JAMA. 2023;330(19):1892‐1902. 37824153 10.1001/jama.2023.12914

[43] Cable CA , Razavi SA , Roback JD , Murphy DJ . RBC transfusion strategies in the ICU: a concise review. Crit Care Med. 2019;47(11):1637‐1644. 31449062 10.1097/CCM.0000000000003985 PMC8319734

[44] Carson JL , Guyatt G , Heddle NM , et al. Clinical practice guidelines from the AABB. JAMA. 2016;316(19):2025‐2035. 27732721 10.1001/jama.2016.9185

[45] Gobatto ALN , Solla D , Brasil S , et al. Progressive hemorrhagic injury and ischemia after severe traumatic brain injury according to hemoglobin transfusion thresholds: a post‐hoc analysis of the transfusion requirements after head trauma trial. Crit Care. 2024;28(1):218. 38961443 10.1186/s13054-024-04981-5 PMC11223415

[46] Guglielmi A , Graziano F , Bogossian EG , Turgeon AF , Taccone FS , Citerio G . Haemoglobin values, transfusion practices, and long‐term outcomes in critically ill patients with traumatic brain injury: a secondary analysis of CENTER‐TBI. Crit Care. 2024;28(1):199. 38877571 10.1186/s13054-024-04980-6 PMC11177426

[47] Gitaari M , Mikolić A , Panenka WJ , Silverberg ND . Diagnostic accuracy of mental health screening tools after mild traumatic brain injury. JAMA Netw Open. 2024;7(7):e2424076. 39042406 10.1001/jamanetworkopen.2024.24076 PMC11267412

[48] Mahncke HW , DeGutis J , Levin H , et al. A randomized clinical trial of plasticity‐based cognitive training in mild traumatic brain injury. Brain. 2021;144(7):1994‐2008. 34312662 10.1093/brain/awab202 PMC8370402

[49] Taccone FS , Badenes R , Rynkowski CB , et al. TRansfusion strategies in acute brain INjured patients (TRAIN): a prospective multicenter randomized interventional trial protocol. Trials. 2023;24(1):20. 36611210 10.1186/s13063-022-07061-7 PMC9825124

