# Supplementary material for: Real‐world efficacy of transfusion with liberal or restrictive strategy in traumatic brain injury
Source: Ann Clin Transl Neurol. 2024 Dec 27;12(1):203–12. doi: 10.1002/acn3.52272 (PMC11752087; doi:10.1002/acn3.52272)

**TABLE OF CONTENTS**

[Supplementary Table 1. The target trial emulation framework of this study 2](#_Toc20967)

[Supplementary Table 2. Algorithms to identify study components from MIMIC-IV. 4](#_Toc8361)

[Supplementary Table 3. Sensitivity analyses of the 28-d risk difference (%) of the main outcomes under various grace time points and restrictive strategy thresholds. 5](#_Toc7696)

[Supplementary Table 4. Doubly robust analyses of the main outcomes of the ETT that compares the efficacy of different transfusion strategies in TBI. 6](#_Toc17035)

[Supplementary Table 5. Subgroup analysis of the risk difference (%) of the main outcomes 7](#_Toc12794)

[Supplementary Figure 1. 10](#_Toc29755)

[Supplementary Figure 2. 11](#_Toc17978)

[Supplementary Figure 3. 1](#_Toc26284)2

[Supplementary Figure 4. 1](#_Toc24570)2

| Supplementary Table 1. The target trial emulation framework of this study | | |
| --- | --- | --- |
| Component | Target trial | Emulation in MIMIC-IV |
| Eligibility | Inclusion Criteria:   - Admitted to hospital due to traumatic brain injury, - Aged 18 years or older, - Hemoglobin lower than 10.0 g/dL within 7 days from ICU admission,   Exclusion Criteria:   - Known pregnant, - Hemorrhagic shock at hospital admission, - Glasgow coma scale (GCS) equal to 3, - Non-reactive pupils bilaterally. | Same as the target trial. |
| Treatment strategies | Liberal red blood cell transfusion strategy (patients will receive transfusion if Hb ≤ 10 g/dL) vs. restrictive transfusion strategy (patients will receive transfusion if Hb ≤ 7.0 g/dL). | Same as the target trial. We allowed a grace period of 24 hours for patients to initiate the treatment in the main analysis. |
| Treatment assignment | Eligible individuals are randomly assigned to one of the two strategies. | Randomization is emulated via cloning individuals and assigning each replicate to a treatment strategy. |
| Follow-up | For each individual, follow-up starts at the time of assignment to a strategy and ends at the occurrence of an outcome, transfer out, or 28 days after the start time, whichever comes first. | Same as the target trial. |
| Primary endpoint | Clinical progression (decrease of GCS by at least 2 points) and all-cause mortality. | Same as the target trial. |
| Causal contrast | Intention-to-treat effect and per protocol effect. | Observational analogue of per protocol effect: effect of adhering to the assigned strategy during follow-up. |
| Statistical analysis | Intention-to-treat analysis.  Per protocol analysis: Individuals are artificially censored when they deviate from their assigned strategy. | Same as per protocol analysis. An expanded dataset was created to include 2 replicates for each included individual and to assign one replicate to each treatment strategy. We adjusted for the baseline covariates and assumed that adjustment for these variables  was sufficient to adjust for informative censoring. |

| Supplementary Table 2. Algorithms to identify study components from MIMIC-IV. | | |
| --- | --- | --- |
| Diagnosis | ICD-9 | ICD-10 |
| Traumatic brain injury | 801.0-801.9; 803.0-804.9; 85-854.1; 951-953; 995.55; 959.01 | S06.1; S06.2; S06.3; S06.5; S06.6; S06.8; S06.9; S06.A; S07.1; S07.8; S07.9 |
| Pregnancy | V22; V23 | Z33; Z34 |
| Hemorrhagic shock | - | R57.1 |
| Hemorrhage | 459.0 | R58 |
| Ischemic heart disease | 410-414 | I20-I25 |
| Multiple injuries | 805-829; 860-897; 959.1-959.9 | S10-S99 |

| Supplementary Table 3. Sensitivity analyses of the 28-d risk difference (%) of the main outcomes under various grace time points and restrictive strategy thresholds. | | | |
| --- | --- | --- | --- |
| Strategy threshold | Grace time | | |
|  | 24 hours | 48 hours | 72 hours |
| **Clinical progression** | |  |  |
| 7.0 g/L | 3.26 (-20.31, 30.10) | 8.88 (-5.07, 30.23) | 7.77 (-2.88, 22.17) |
| 7.5 g/L | 6.75 (-16.18, 32.68) | 9.63 (-4.34, 30.87) | 9.56 (-2.13, 22.77) |
| 8.0 g/L | 5.16 (-16.78, 31.61) | 6.36 (-5.88, 29.51) | 8.00 (-1.49, 21.24) |
| **All-cause mortality** | |  |  |
| 7.0 g/L | -2.97 (-12.65, 16.92) | -1.78 (-10.19, 10.92) | 1.63 (-7.30, 12.16) |
| 7.5 g/L | -3.21 (-12.50, 16.23) | -1.55 (-9.91, 11.42) | 1.97 (-6.86, 12.69) |
| 8.0 g/L | -3.13 (-12.02, 15.90) | -1.38 (-9.79, 10.83) | 2.00 (-6.54, 12.00) |

| Supplementary Table 4. Doubly robust analyses of the main outcomes of the ETT that compares the efficacy of different transfusion strategies in TBI. | | |
| --- | --- | --- |
| Time points | Cumulative risk difference (%) | |
|  | Primary analysis | Doubly robust approach |
| **Clinical progression** | | |
| 3-day | 2.91 (-6.53, 10.15) | 1.12 (-1.01, 3.77) |
| 7-day | 4.59 (-10.83, 14.31) | 1.78 (-1.11, 5.50) |
| 14-day | 5.40 (-15.20, 20.15) | 2.27 (-1.29, 8.07) |
| 28-day | 3.26 (-20.31, 30.10) | 2.31 (-3.78, 20.82) |
| **All-cause mortality** | | |
| 3-day | -5.02 (-7.51, -2.40) | -0.87 (-1.40, -0.35) |
| 7-day | -6.27 (-11.22, -3.00) | -1.01 (-2.11, -0.25) |
| 14-day | -2.34 (-11.80, 14.56) | -0.13 (-2.13, 2.57) |
| 28-day | -2.97 (-14.03, 19.61) | 0.06 (-2.08, 4.72) |
| Abbreviations: ETT, emulated target trial; TBI, traumatic brain injury. | | |

| Supplementary Table 5. Subgroup analysis of the risk difference (%) of the main outcomes | | | | |
| --- | --- | --- | --- | --- |
| Group | Time points | | | |
|  | 3-day | 7-day | 14-day | 28-day |
| **Clinical progression** | | | | |
| Sex |  |  |  |  |
| Male | 1.27 (-10.80, 12.35) | -0.41 (-20.05, 14.23) | -2.82 (-26.48, 17.54) | -5.34 (-26.50, 35.20) |
| Female | 7.11 (-4.38, 17.94) | 6.84 (-11.09, 23.70) | 6.14 (-14.94, 38.41) | 8.40 (-13.32, 47.95) |
| Age |  |  |  |  |
| ≥65 | 2.56 (-15.32, 30.63) | -1.25 (-28.52, 37.63) | -2.68 (-30.06, 56.9) | 41.05 (-3.94, 56.71) |
| <65 | -3.53 (-10.53, 5.33) | -4.74 (-15.85, 6.61) | -4.42 (-23.6, 12.81) | -1.62 (-22.91, 24.98) |
| GCS |  |  |  |  |
| >12 | 4.66 (-3.70, 11.24) | 5.60 (-5.42, 15.79) | 5.00 (-7.90, 21.80) | 1.41 (-17.70, 31.05) |
| ≤12* | NA | NA | NA | NA |
| Lactate |  |  |  |  |
| <2 | -4.82 (-16.42, 13.2) | -12.95 (-31.7, 17.1) | -18.86 (-38.71, 21.16) | -17.10 (-36.59, 35.86) |
| ≥2 | 0.73 (-6.60, 10.06) | 6.40 (-3.21, 22.34) | 11.83 (-0.70, 30.82) | 5.92 (-18.94, 31.75) |
| Vasopressor |  |  |  |  |
| Yes | 0.93 (-8.09, 9.31) | 4.12 (-10.49, 13.34) | 3.59 (-12.90, 15.64) | -1.82 (-17.05, 12.58) |
| No | 6.87 (-5.87, 17.46) | 7.53 (-10.80, 19.59) | 8.14 (-9.37, 29.14) | 18.30 (-7.08, 55.67) |
| MAP |  |  |  |  |
| <70 | 3.48 (-6.13, 20.87) | 2.32 (-9.82, 18.96) | 3 (-12.10, 29.31) | 3.47 (-11.31, 56.36) |
| ≥70 | 1.17 (-7.07, 10.22) | 1.05 (-12.73, 14.62) | 0.58 (-17.07, 20.95) | 0.35 (-22.47, 42.04) |
| Ischemic heart disease | | | | |
| Yes | -1.13 (-14.18, 48.79) | -0.79 (-21.87, 50.64) | 52.84 (-12.65, 65.5) | 52.56 (-13.17, 65.34) |
| No | 3.24 (-5.57, 14.96) | 3.45 (-9.64, 16.63) | 2.67 (-17.05, 18.59) | 1.00 (-18.53, 24.60) |
| Multiple injuries | | | | |
| Yes | 5.82 (-4.85, 14.3) | 10.49 (-1.33, 20.96) | 12.10 (-8.23, 27.48) | 6.21 (-19.16, 30.8) |
| No | -7.90 (-27.05, 29.17) | -17.93 (-37.36, 25.67) | -24.50 (-45.58, 25.94) | 8.03 (-37.26, 50.90) |
| **All-cause mortality** | |  |  |  |
| Sex |  |  |  |  |
| Male | -5.18 (-8.23, 0.28) | -6.80 (-12.45, 0.76) | -2.69 (-13.91, 22.50) | -7.21 (-15.80, 34.69) |
| Female | -6.20 (-8.32, -3.06) | -8.39 (-11.16, -4.06) | -3.65 (-16.09, 25.50) | -2.90 (-19.56, 22.89) |
| Age |  |  |  |  |
| ≥65 | -7.03 (-9.94, -4.58) | -9.15 (-16.68, -3.28) | 12.13 (-15.41, 65.9) | 11.21 (-19.13, 71.67) |
| <65 | -1.96 (-4.29, -0.16) | -3.27 (-6.97, -0.48) | -4.29 (-8.94, -0.27) | -4.70 (-10.05, -0.29) |
| GCS |  |  |  |  |
| >12 | -4.42 (-6.93, -1.89) | -5.53 (-9.29, -2.23) | -4.42 (-11.49, 9.11) | -6.18 (-13.40, 8.13) |
| ≤12* | NA | NA | NA | NA |
| Lactate |  |  |  |  |
| <2 | -3.76 (-5.65, -1.70) | -6.78 (-10.72, -3.14) | -2.74 (-15.44, 38.43) | -2.09 (-18.47, 48.33) |
| ≥2 | -3.52 (-8.28, 1.72) | -7.55 (-13.80, 0.72) | -11.19 (-17.91, -1.18) | -14.75 (-21.30, -3.29) |
| Vasopressor |  |  |  |  |
| Yes | -4.90 (-9.27, 2.00) | -9.01 (-15.74, -0.06) | -13.00 (-22.41, -4.22) | -14.53 (-25.75, -5.29) |
| No | -3.81 (-4.99, -2.30) | -6.67 (-8.37, -3.61) | 6.97 (-9.30, 27.76) | 6.94 (-12.09, 32.06) |
| MAP |  |  |  |  |
| <70 | -4.60 (-9.59, -1.58) | -9.27 (-16.39, -5.55) | -13.88 (-21.72, -8.54) | -16.49 (-24.51, -10.31) |
| ≥70 | -4.49 (-6.3, -1.94) | -5.49 (-9.4, -1.37) | 5.23 (-11.22, 28.64) | 5.58 (-14.26, 31.74) |
| Ischemic heart disease | | | |  |
| Yes | -4.89 (-8.66, -0.10) | -12.31 (-16.88, -4.28) | -21.02 (-26.63, -9.76) | -25.99 (-33.39, -14.94) |
| No | -5.06 (-7.96, -1.53) | -5.35 (-10.43, -0.75) | 2.53 (-9.22, 21.75) | 3.23 (-10.47, 26.83) |
| Multiple injuries | | | | |
| Yes | -2.51 (-5.66, 1.58) | -3.01 (-7.37, 3.45) | -3.05 (-10.23, 7.74) | -4.45 (-11.50, 6.54) |
| No | -8.39 (-11.79, -5.44) | -14.37 (-18.52, -10.12) | 8.12 (-21.62, 78.08) | 9.5 (-25.65, 75.89) |
| * The subgroup included too few samples to provide a valid bootstrap estimate.  Abbreviations: GCS, Glasgow coma scale; MAP, mean arterial pressure. | | | | |

Supplementary figure legends

Supplementary Figure 1. Flow diagram of study individual selection. Abbreviations: TBI, traumatic brain injury; Hb, hemoglobin; GCS, Glasgow coma scale.


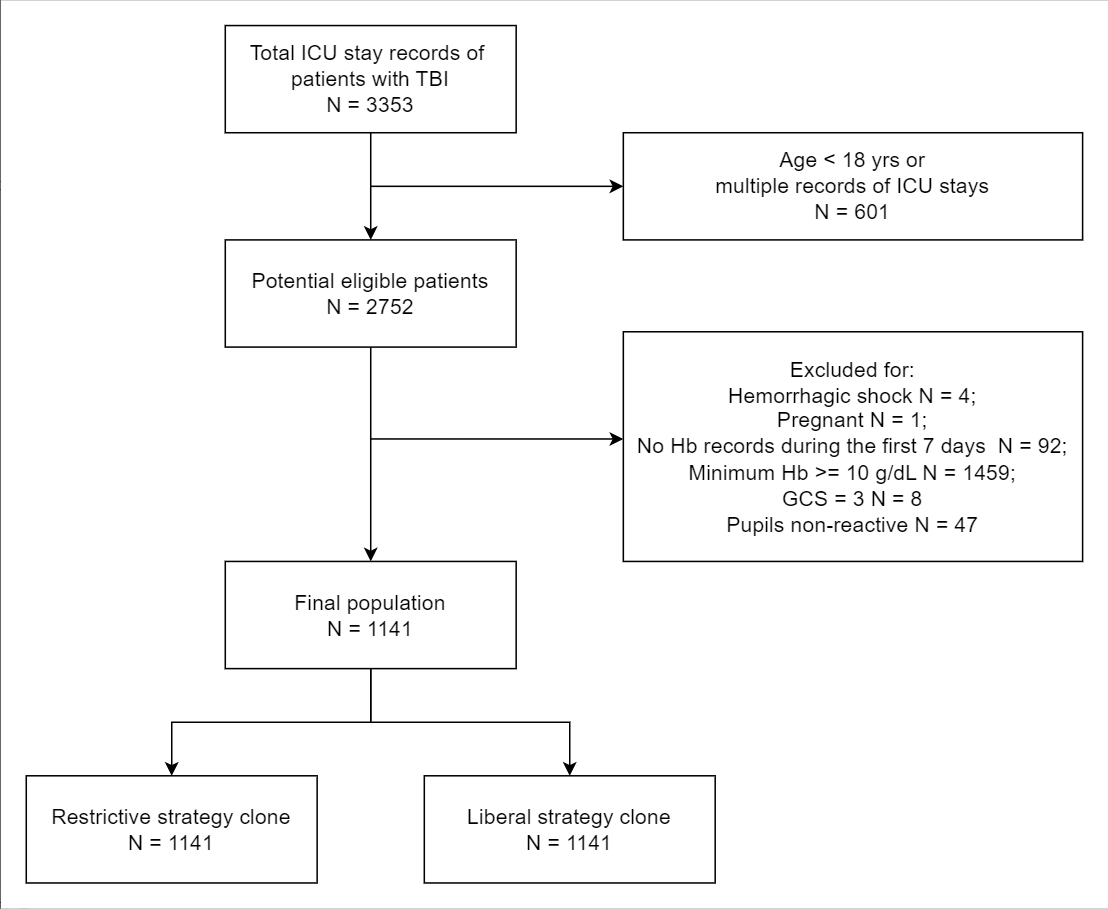


Supplementary Figure 2. Causal directed acyclic graph to represent the causal relationship between red blood cell transfusion strategy and mortality. The selected confounders are marked in red color. Abbreviations: WBC, white blood cell; PLT, platelet; BUN, blood urea nitrogen; GCS, Glasgow coma scale; MAP, mean arterial pressure; HGB, hemoglobin; LAC, lactate; HR, heart rate; RR, respiratory rate.


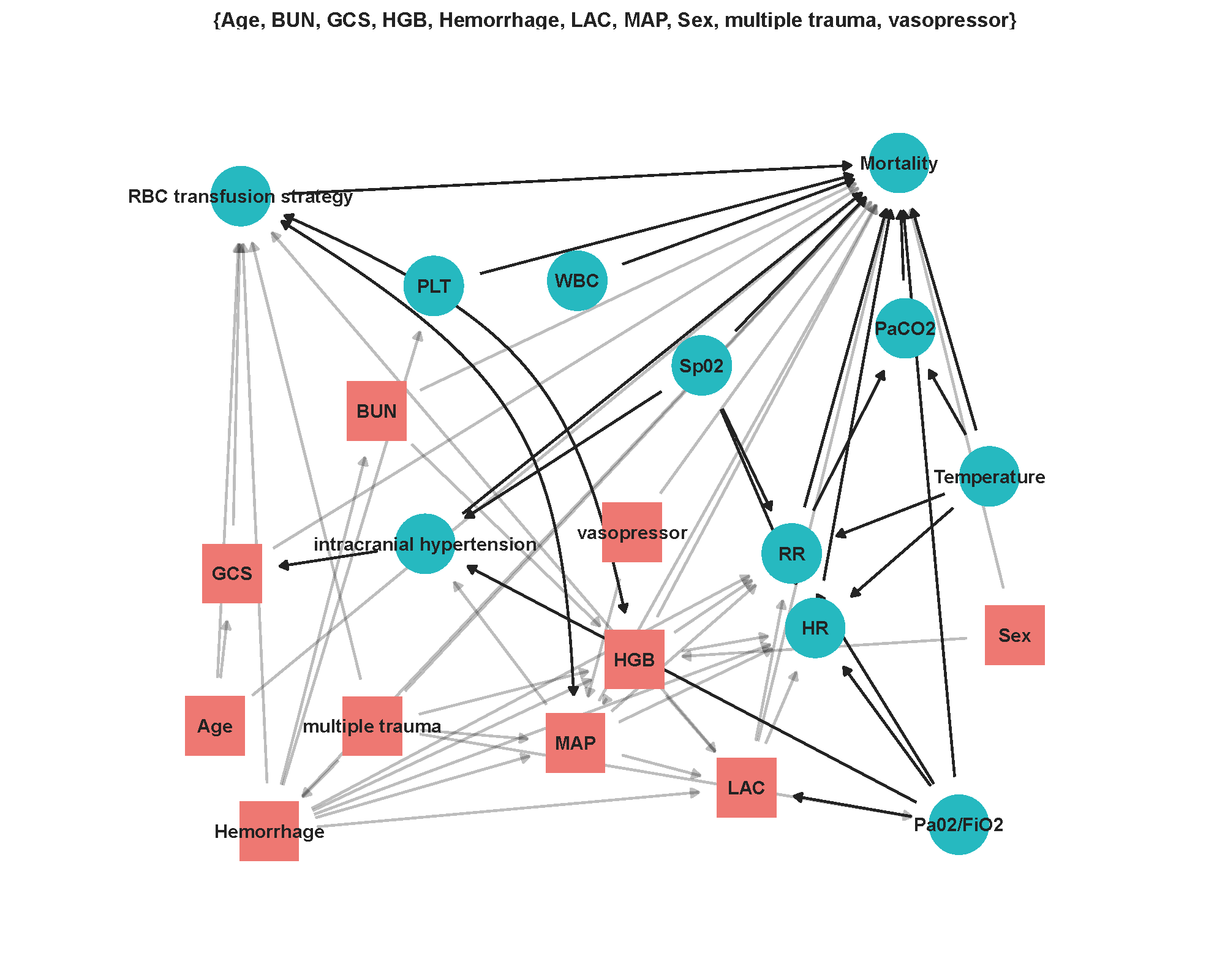


Supplementary Figure 3. Standardized mean differences of the confounders in uncensored population before and after inverse probability weighting. Abbreviations: HGB, hemoglobin; LAC, lactate; MT, multiple trauma; ICP, intracranial pressure; MAP, mean arterial pressure; GCS, Glasgow coma scale; BUN, blood urea nitrogen; IHD, ischemic heart disease.


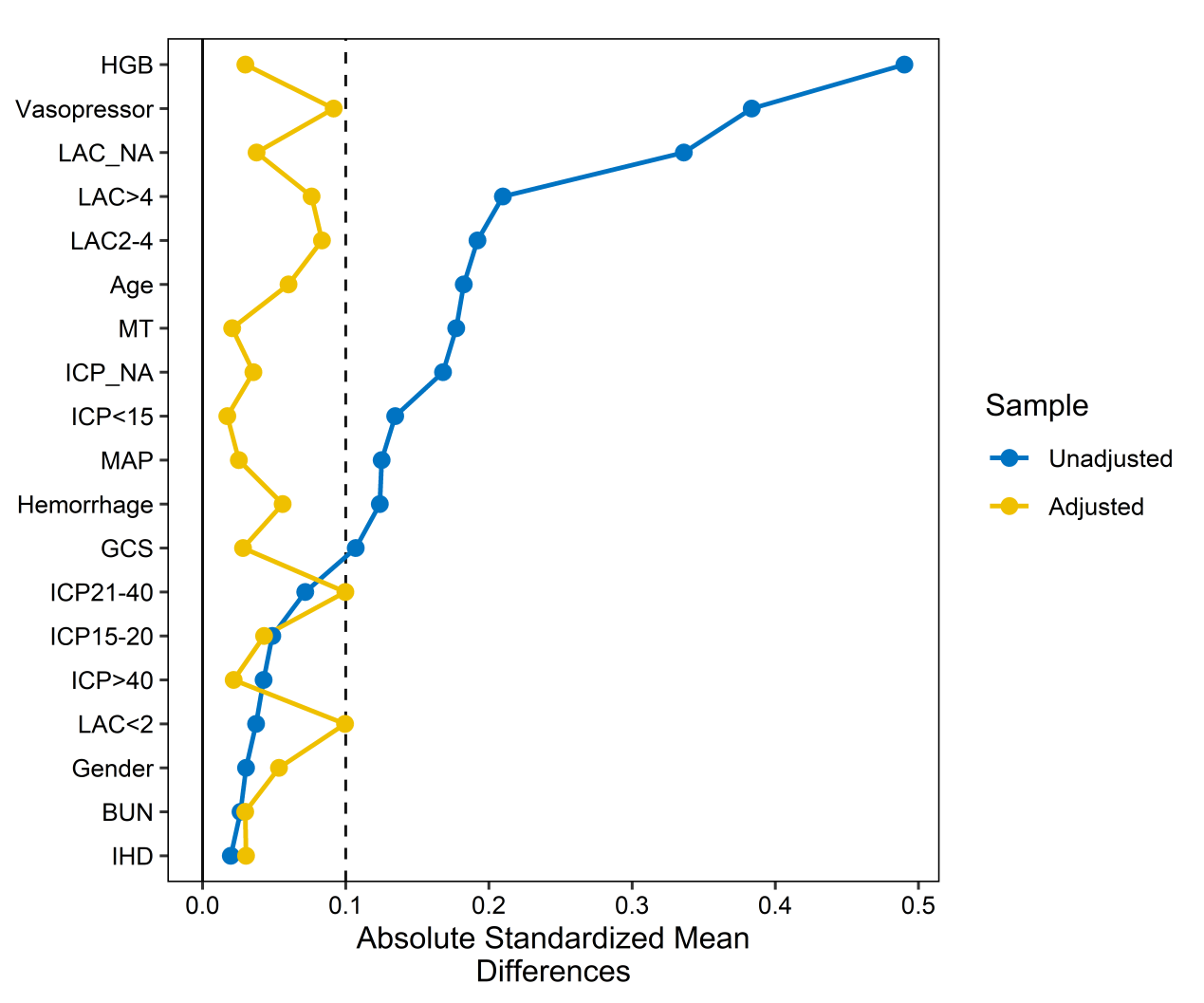


Supplementary Figure 4. Sensitivity analyses of the main outcomes under various grace time points and restrictive strategy thresholds. (A) Clinical progression; (B) All-cause mortality.


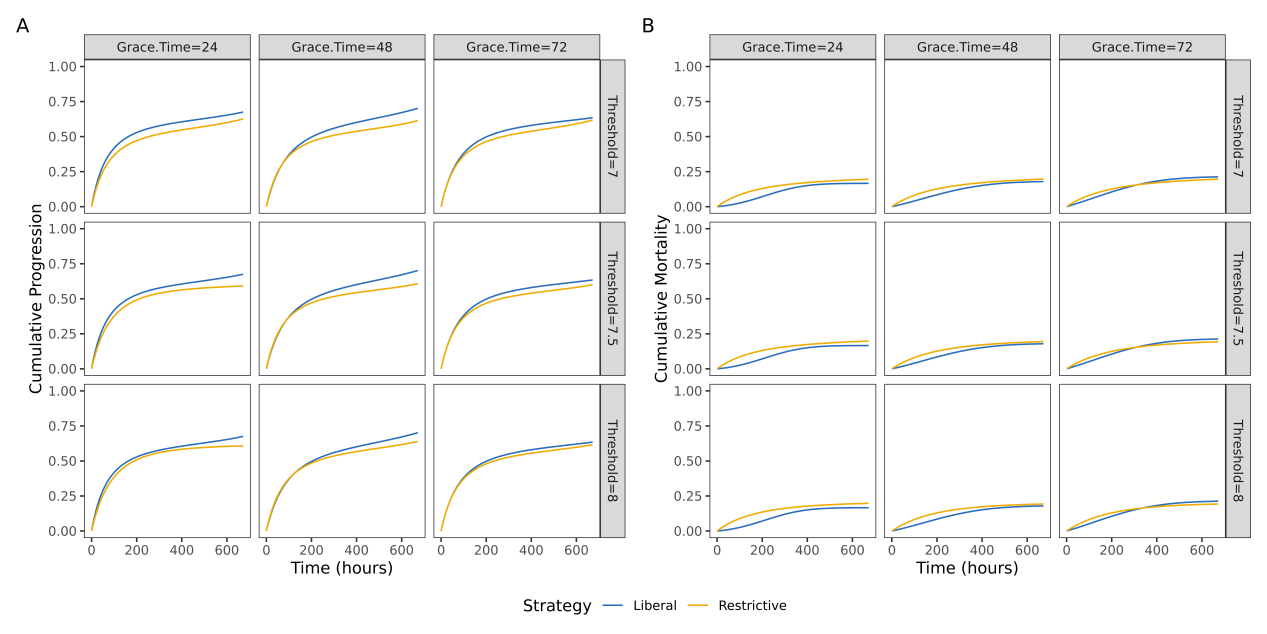

Supplement: Supplementary file 1 — Data S1. [file ACN3-12-203-s001.docx]
